# Simultaneous invasive and non-invasive recordings in humans: A novel Rosetta stone for deciphering brain activity

**DOI:** 10.1016/j.jneumeth.2024.110160

**Published:** 2024-05-09

**Authors:** Andrea Pigorini, Pietro Avanzini, Andrei Barborica, Christian-G. Bénar, Olivier David, Michele Farisco, Corey J. Keller, Alfredo Manfridi, Ezequiel Mikulan, Angelique C. Paulk, Nicolas Roehri, Ajay Subramanian, Serge Vulliémoz, Rina Zelmann

**Affiliations:** a Department of Biomedical, Surgical and Dental Sciences, Università degli Studi di Milano, Milan, Italy; b UOC Maxillo-facial Surgery and dentistry, Fondazione IRCCS Cà Granda, Ospedale Maggiore Policlinico, Milan, Italy; c Institute of Neuroscience, Consiglio Nazionale delle Ricerche, Parma, Italy; d Physics Department, University of Bucharest, Bucharest, Romania; e Aix Marseille Univ, Inserm, U1106, INS, Institut de Neurosciences des Systèmes, Marseille, France; f Centre for Research Ethics and Bioethics, Department of Public Health and Caring Sciences, Uppsala University, P.O. Box 256, Uppsala, SE 751 05, Sweden; g Science and Society Unit Biogem, Biology and Molecular Genetics Institute, Via Camporeale snc, Ariano Irpino, AV 83031, Italy; h Department of Psychiatry & Behavioral Sciences, Stanford University Medical Center, Stanford, CA 94305, USA; i Wu Tsai Neurosciences Institute, Stanford University Medical Center, Stanford, CA 94305, USA; j Veterans Affairs Palo Alto Healthcare System, and the Sierra Pacific Mental Illness, Research, Education, and Clinical Center (MIRECC), Palo Alto, CA 94394, USA; k Department of Pathophysiology and Transplantation, Università degli Studi di Milano, Milan, Italy; l Department of Health Sciences, Università degli Studi di Milano, Milan, Italy; m Department of Neurology and Center for Neurotechnology and Neurorecovery, Massachusetts General Hospital and Harvard Medical School, Boston, MA, USA; n EEG and Epilepsy Unit, Dpt of Clinical Neurosciences, Geneva University Hospitals and University of Geneva, Switzerland

**Keywords:** IEEG, Simultaneous recordings, Invasive and non-invasive, Hd-EEG, MEG

## Abstract

Simultaneous noninvasive and invasive electrophysiological recordings provide a unique opportunity to achieve a comprehensive understanding of human brain activity, much like a Rosetta stone for human neuroscience. In this review we focus on the increasingly-used powerful combination of intracranial electroencephalography (iEEG) with scalp electroencephalography (EEG) or magnetoencephalography (MEG). We first provide practical insight on how to achieve these technically challenging recordings. We then provide examples from clinical research on how simultaneous recordings are advancing our understanding of epilepsy. This is followed by the illustration of how human neuroscience and methodological advances could benefit from these simultaneous recordings. We conclude with a call for open data sharing and collaboration, while ensuring neuroethical approaches and argue that only with a true collaborative approach the promises of simultaneous recordings will be fulfilled.

## 1. Introduction

In 1799, a French captain named Pierre-François Bouchard discovered a block of rock in the port city of Rosetta, in the Nile delta. This discovery changed the way archaeologists understood ancient history forever. The rock, known as the Rosetta Stone, bore inscriptions in three different languages: ancient Greek, demotic Egyptian, and Egyptian hieroglyphs. By simultaneously presenting the same text in three different languages, with one well-known to scholars (ancient Greek), the Rosetta Stone allowed the translation of many hieroglyphs, forever changing the study of archaeology. Modern neuroscience finds itself in a similar situation as archaeology in the 1800 s: the human brain speaks in hieroglyphs, and there is no way to fully understand it because of a lack of deciphering methods. In this review, we argue that simultaneous multiscale electrophysiological recordings can act as a Rosetta stone for neuroscience by providing a comprehensive understanding of human brain activity.

Since the human brain is safely enclosed within the skull, the neural correlates of human behavior and disease have been studied primarily indirectly, by recording neuronal activity from outside the skull. One of the most commonly used tools for non-invasive recordings of brain activity is the electroencephalogram (EEG). First used in humans in 1929 by Hans Berger (Berger, 1929), the EEG captures the electrical activity of neurons in the human cerebral cortex through conducting electrodes placed on the scalp. With sub-millisecond resolution, EEG measures voltage deflections primarily from post-synaptic potentials. More recently, magnetoencephalography (MEG) was developed in 1968 to measure the magnetic counterpart of neuronal activity (Cohen, 1972). While MEG is relatively insensitive to the medium in which magnetic fields propagate as it is not spatially smeared by the low skull conductivity, it is less sensitive than EEG to radial sources (Ahlfors et al., 2010). Thus, the complement of EEG and MEG can capture multiple sources of neuronal activity from outside of the brain (Lopes da Silva, 2013).

In human neuroscience, functional magnetic resonance imaging (fMRI) has allowed a detailed volumetric brain-wide understanding of cognition and development, but with limited temporal resolution (Glover, 2011; Ogawa et al., 1990). Combining EEG and fMRI yields high spatial and temporal resolution, all within the non-invasive domain (Bénar et al., 2006; Huster et al., 2012). Still, to obtain direct insight into human brain functioning, recording electrophysiology from inside the human brain, as it is done with animal models, is closer to the local neuronal activity.

Recording human brain activity from inside the skull is much more challenging and less frequent, but certain unique situations provide access to direct brain recordings in humans, such as drug-resistant epilepsy. Patients with epilepsy that do not respond to anti-epileptic drugs and with a suspected focal seizure onset zone may undergo surgical removal of the pathological portions of the brain responsible for seizures (Luders, 2008) or more recently chronic neuromodulation implantation (Fisher et al., 2010; Jarosiewicz and Morrell, 2021; Morrell and RNS System in Epilepsy Study Group, 2011; Salanova et al., 2021). For many of these surgical candidates, non-invasive pre-surgical evaluation is sufficient to identify the epileptic generator. For more complicated cases, however, to precisely identify the epileptic brain tissue and minimize collateral damage resulting from surgical resection, intracranial EEG recordings are obtained by inserting electrodes in or placing them on the patient’s brain prior to the surgical intervention (McGonigal et al., 2007; Olivier et al., 2012; Schomer and Lopes da Silva, 2017). This invasive examination involves the insertion of thin depth electrodes (stereo-electroencephalography, SEEG) or subdural grids and strip electrodes on the surface of the brain (Electrocorticography, ECoG), which are then used to directly record the electrical activity of populations of neurons from human brain tissue (both will be referred as intracranial EEG - iEEG). Patients remain in the hospital, semi-chronically implanted from a few days up to three weeks, to continuously acquire their iEEG. This allows for a better understanding of the network involved in occurrence of interictal and ictal epileptic activity and to delineate planned surgical removal. In addition, this invasive examination allows for the identification of “eloquent” cerebral areas responsible for essential functions such as vision, hearing, movement, and language, which cannot be affected during surgery to avoid functional deficits.

ECoG and SEEG were independently introduced in the 1950 s (Schijns et al., 2015) in Montreal, Canada (Penfield and Jasper, 1954) and France (Talairach et al., 1962). ECoG subdural grids require a craniotomy and were initially used acutely in the operating room (Penfield and Jasper, 1954) while SEEG could be implanted with a stereotaxic frame to guide electrode insertion (Talairach et al., 1962). In the early 1990 s, frameless image-based SEEG implantations were adopted (Doshi et al., 1995; Hashizume et al., 1997; Olivier et al., 1994), allowing for oblique trajectories and simplifying the procedure. iEEG has been routinely acquired for the pre-surgical evaluation of medically refractory epileptic patients since then. Recently, the number of centers that implant SEEG has considerably increased (Gavvala et al., 2022; Isnard et al., 2018). This is likely due to technological advances, such as robotic-aided implantations (González-Martínez et al., 2016), and to a shift in centers that used to implant subdural grids and now implanting depth-electrodes (Gavvala et al., 2022). Since depth electrodes are inserted into the brain through small holes in the skull and do not require a craniotomy, the clinical morbidity is lower (4.8% for SEEG vs. 15.5% for grids, in (Yan et al., 2019)) at comparative effectiveness (Jehi et al., 2021; Tandon et al., 2019).

Although iEEG recordings are performed to localize seizure focus, they also sample from brain regions located outside of the seizure-generating regions and thus can provide a unique window into the human brain. This data can be retrospectively mined to both improve our understanding and treatment of epilepsy, and to study the neurophysiological mechanisms underlying the functioning of the human brain (Jobst et al., 2020; Johnson et al., 2020; Parvizi and Kastner, 2018). While iEEG recordings deliver unique insight, significant spatial sampling heterogeneity exists across patients and translation of findings from these unique brain recordings to other populations is difficult. This limitation begs the question: How can we relate whole brain noninvasive recordings from scalp-EEG/MEG that can be obtained from any individual with intracerebral recordings which more directly, though sparsely, capture brain activity in patients with epilepsy?

To bridge the gap between these two modalities, and to delve deeper into the neuronal mechanisms underlying human-specific brain properties, an ideal scenario is to simultaneously record from inside (iEEG) and outside (EEG/MEG) the human parenchyma. These simultaneous recordings would allow for interpretation of noninvasive recordings (EEG/MEG) in light of what is directly observable from the cerebral cortex with invasive recordings (iEEG). This combination can help validate non-invasive methods in accurate identification of epileptogenic areas, while allowing for the transfer of neuroscientific discoveries during iEEG recordings to non-invasive experimental setups. This narrative review focuses on studies that employed SEEG and ECoG and not on ones using Deep Brain Stimulation (DBS). For DBS studies, see for example (Harmsen et al., 2018; Litvak et al., 2021). In Section 2, we discuss the technical challenges of combining MEG/EEG and intracranial EEG and offer practical recommendations. In Section 3, we describe clinical applications for epilepsy. In Section 4, we illustrate applications to cognitive neuroscience research and discuss promising future directions of combining invasive and non-invasive technologies. Finally, in Section 5, we discuss other spatial scales, ethical considerations, and the importance of sharing these unique datasets.

## 2. How to combine invasive and non-invasive recordings: from experimental setup to data analysis

**Fig. 1.**
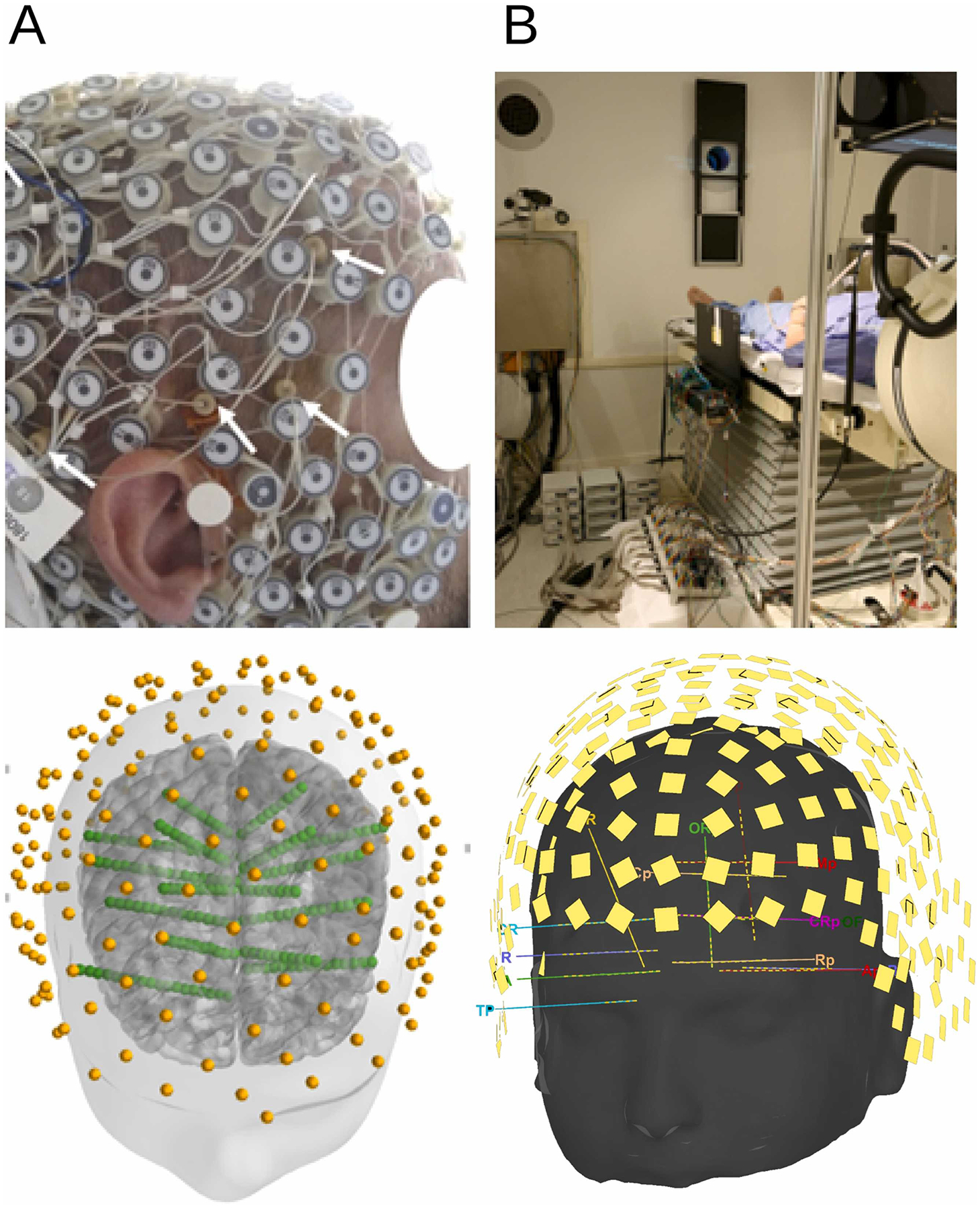
Intracranial correlates of interictal epileptic activity. (a) Simultaneous hd-EEG and stereo-EEG (adapted from (De Stefano et al., 2022; Mikulan et al., 2020)). (b) Simultaneous MEG and stereo-EEG (adapted from (Velmurugan et al., 2022)).

Combining invasive and non-invasive recordings in humans presents numerous technical challenges that need to be addressed throughout the acquisition setup and data analysis stages. These challenges are particular to the kind of devices/methods used to record brain activity and apply to both invasive and noninvasive recordings. Most recent simultaneous studies have used SEEG depth-electrodes. SEEG is less invasive than ECoG, there is no silastic grids that may distort the scalp signal (von Ellenrieder et al., 2014), and, most importantly, SEEG leaves the skull almost intact thus limiting possible infections (Katz and Abel, 2019; Tandon et al., 2019). Simultaneous non-invasive scalp recordings with standard individual electrode positioning, following the international 10–20 system (Fig. 1A) are clinically used in some centers during the patient’s intracranial monitoring primarily for sleep staging. This could be leveraged for research purposes (as in Abramovici et al., 2018; Tao et al., 2005; Zelmann et al., 2014) and has the advantage of being long-term. To obtain higher spatial resolution up to 256 channels for a limited time (up to a few hours) high-density EEG (hd-EEG, Fig. 1B)(De Stefano et al., 2022; Mikulan et al., 2020) or MEG, (Fig. 1C) (Badier et al., 2017; Velmurugan et al., 2022) provide a direct relation to non-invasive paradigms. In a few cases, it was feasible to combine all three modalities, demonstrating the feasibility of iEEG-EEG-MEG recordings (Dubarry et al., 2014; Gavaret et al., 2016). In addition, several studies have demonstrated the technical possibility to simultaneously record cortical activity with MEG/EEG and deep nuclei in patients undergoing deep brain stimulation procedures (e.g., Beck et al., 2018; Bock et al., 2013; Hirschmann et al., 2011; Litvak et al., 2010; Norton et al., 2012; van Wijk et al., 2017).

Regardless of the specific devices and setup used for invasive and non-invasive recordings, achieving successful integration between the systems while ensuring the safety of the patients requires careful consideration of several key factors regarding hardware compatibility, synchronization, preprocessing, analysis, and interpretation of the combined data. Below, we summarize these technical challenges.

### Coverage and Safety.

The first and most crucial aspect in combining invasive and non-invasive recordings lies in the experimental setup and the minimization of possible safety issues. Arrangement of non-invasive electrodes/coils on the scalp in the presence of an intracranial implant (both with depth electrodes and grids) is not trivial. Specifically, the challenge lies in achieving optimal coverage and alignment of the electrodes while minimizing the risk of infection, tissue damage, or adverse effects. For example, in the case of MEG, the post-operative bandage wrapped around the head and the SEEG insertion screws can result in increased volume, possibly preventing the head from fitting the MEG helmet (Badier et al., 2017). In the case of hd-EEG, instead, the bandage should be removed to fit the EEG net system on the participants’ head, leading to possible safety issues due to the mechanical interaction of the two - wired - systems and the extra risk of infection. However, these aspects can be carefully addressed with specific protocols, such as sterilization of the EEG cap, disinfecting the skin after removal of the protective bandage, and disinfecting again after the EEG cap was removed (see e.g., (Mikulan et al., 2020) for more details).

### Temporal alignment.

Ensuring accurate synchronization of data becomes crucial. Precise temporal alignment between invasive and non-invasive recordings is necessary to establish meaningful relationships between the signals. The best solution consists of using the same amplifier for the acquisition of both non-invasive and invasive signals, which is currently only possible in the case of low-density EEG (Barborica et al., 2021). When this is not the case, as in current MEG and hd-EEG studies, several approaches can be used to achieve synchronization. For example, aligning spontaneous recordings requires external triggers that mark the start and end of data acquisition across modalities or that continuously send evenly spaced markers (i.e., TTL square signals, (De Stefano et al., 2022)) or identifiable markers spaced with randomized jittered time windows to give each time point a unique identifier. Along the same line, to align event-related signals, it is ideal to send identical markers to both acquisition systems at the onset of the events (Barborica et al., 2023; Mikulan et al., 2020; Parmigiani et al., 2022). This becomes increasingly important in the presence of possible clock drifts between the acquisition computers, which is crucial for successful synchronization of long-lasting recording (Bénar and Badier, 2019) spontaneous activity (e.g., sleep, epileptic activity, and natural behaviour). One option to monitor the clock drift over longer recording durations is to apply a continuous reference signal (e.g. a 50 Hz sine wave) to EEG inputs on the two systems, calculate the lag of the cross-correlation between the common reference signal across time and apply necessary timestamp corrections (Barborica et al., 2021).

### Spatial alignment.

To make the results of invasive and non-invasive recordings comparable, they need to be aligned not only in time but also in the spatial domain. The location of the intracerebral contacts can be recovered by co-registration of MRI anatomical image and CT/MRI post-implantation scan (Davis et al., 2021; Li et al., 2019; Medina Villalon et al., 2018; Narizzano et al., 2017; Soper et al., 2023; Zelmann et al., 2023). In terms of surface signal, the exact position of the scalp contacts/coils must be carefully determined. This can be achieved through standard procedures such as using 10–20 placement (Barborica et al., 2021) or by digitizing them with respect to the patients’ MRI/CT images (Mikulan et al., 2020; Parmigiani et al., 2022). In contrast to standard scalp EEG analysis, which can utilize templates of electrode positions to compare data across different participants, the position of scalp contacts can vary significantly in simultaneous recordings due to the constraints imposed by the fixation system of the intracerebral implant (Mikulan et al., 2020).

Finally, when considering spatial alignment methods for scalp EEG recordings, during data acquisition the choice between using a pen-like pointer such as a Polhemus system or a camera-based system should be carefully considered (Michel and Murray, 2012). Pen-like pointers provide reasonably accurate spatial alignment but may be susceptible to errors due to movement artifacts and calibration inaccuracies (Burgess and Gruzelier, 1997). Camera-based systems offer a safer approach (because it does not include touching the scalp with the pen) with greater flexibility and comfort for the participant, although they may require careful setup and calibration (Michel and Murray, 2012). Recent advancements in EEG technology, such as the integration of inertial measurement units (IMUs) into EEG caps or headsets, offer alternative approaches for spatial alignment and motion tracking (Gwin et al., 2011).

### Preprocessing.

The preprocessing of simultaneously recorded signals, in general, can be performed mostly separately for the invasive and non-invasive data by using standard preprocessing pipelines - including algorithms for the automatic detection of electrical artifacts, pathological or physiological activity (e.g., Delphos for intracerebral data, (Roehri et al., 2016). An additional step of preprocessing that should be considered is the combined epoch rejection. Indeed, some artifacts can be present only in intracerebral data but not visible on the scalp EEG and vice versa. Thus, depending on the aim of the data collection and particularly for physiology and cognitive studies in which epileptic activity should be excluded from the analysis, joint epoch rejection is needed. Alternatively, the analysis can be performed on the average of different epochs (Parmigiani et al., 2022).

### Analysis.

After preprocessing, the integration of invasive and non-invasive signals presents another technical challenge. Although correlating the two modalities is not trivial it is worth to obtain a comprehensive understanding of brain physiology. Initial studies compared the results obtained from the two modalities analyzed separately, for example in terms of time-frequency content (Dalal et al., 2009; Dubarry et al., 2014; Rampp et al., 2010) or results of source localization (Wennberg et al., 2011). However, the real power of simultaneous recordings lies in the correlation across time of the non-invasive signals with the invasive one (Dubarry et al., 2014) or in a joint analysis of the two modalities (Gavaret et al., 2016). In particular the relation to signals from deep structures is challenging, as the field from a dipolar source decreases as the square of the distance and some brain structures, such as the amygdala, may have a closed electric field. Spatial filtering technique or independent component analysis (ICA) can potentially help disentangling signals seen at the surface and thus facilitate the comparison with depth signals (Coelli et al., 2023; López-Madrona et al., 2022; Pizzo et al., 2019). For instance, in MEG, ICA was used to separate the different parts of the epileptic network, with some components correlating only with deep SEEG contacts, confirming the possibility to see deep mesial sources from the surface. The deep components had a smaller amplitude at the surface, but a distinctive MEG topography (Pizzo et al., 2019). The usefulness of ICA for capturing deep activity was also confirmed in a later study on simultaneous scalp EEG-iEEG (Ternisien et al., 2023). In addition, multivariate statistical methods, such as multivariate pattern analysis (MVPA; Barborica et al., 2023) or machine learning algorithms (Li et al., 2021; Abou Jaoude et al., 2022), could help identify patterns and relationships that may not be apparent when analyzing each modality separately, revealing hidden associations, causal relationships, and gain deeper insights into the underlying neural processes.

Overall, combining invasive and non-invasive recordings in humans entails several technical challenges that span from the experimental setup to data analysis. By addressing these technical issues, researchers can make significant strides in the development of new methods for the clinical and surgical treatment of drug-resistant epilepsy and demonstrating the clinical utility of performing simultaneous invasive and non-invasive recordings (see Section 3). They can also gain new insights into cognitive and physiological phenomena that cannot be addressed by invasive or non-invasive methods alone (see Section 4).

## 3. Clinical applications: identifying the seizure onset zone with simultaneous recordings

With the aim of gaining a comprehensive understanding of epileptic activity, the motivation for recording simultaneously scalp and iEEG in the clinical setting is multifaceted to help improve diagnosis, treatment, and prognosis. The first clinical advantage of simultaneous scalp and intracranial recording is to provide the clinical team with the knowledge of how the iEEG findings correspond to the non-invasive interictal and ictal scalp EEG activity previously recorded. This includes the localization of activity to deep regions and the propagation of epileptic seizures. A second clinical advantage is to have coverage of regions not recorded by the intracranial electrodes, such as other lobules or the other hemisphere.

A translational research motivation of clinical importance is to characterize the network correlates of scalp patterns for the identification of new scalp biomarkers and the differentiation of patterns arising from pathological or physiological brain activity. Finally, another important motivation is methodological: i) to better understand the visibility of pathological discharges observed in EEG or MEG, ii) to enhance the future utilization of non-invasive electrophysiology in planning intracranial electrode implantation, and iii) to develop and fine-tune signal processing methods for improved detection of subtle activities, whether originating deep in the brain or involving small cortical areas.

### 3.1. Detection of epileptic activity with scalp EEG

**Fig. 2.**
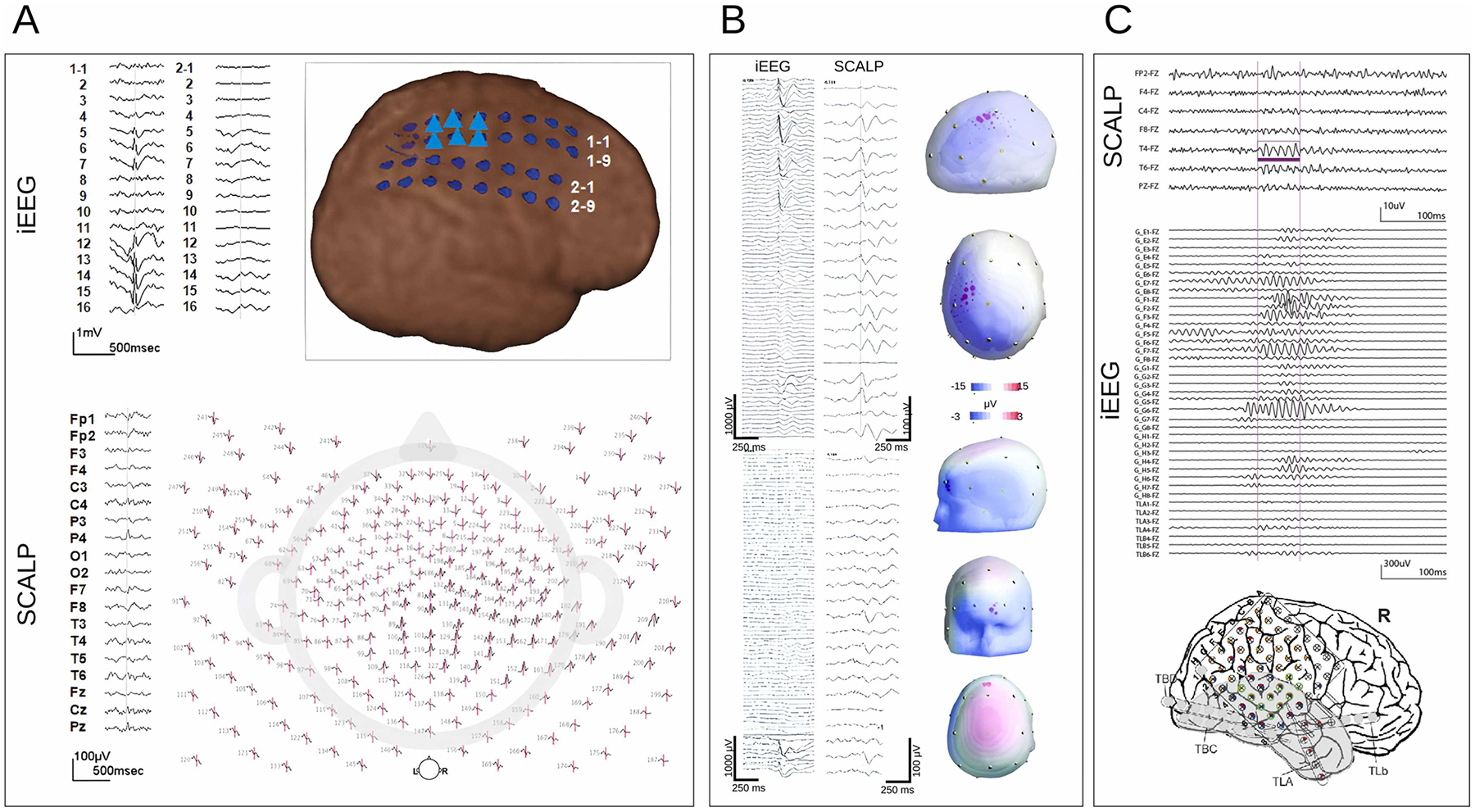
Intracranial correlates of scalp EEG transient to assess their epileptic relevance. (a) Simultaneous scalp hd-EEG and intracranial IEDs (adapted from (Yamazaki et al., 2013)). (b) Simultaneous scalp 10–20 system and intracranial IEDs of parietal (top) and orbitofrontal (bottom) origins (adapted from (Ramantani et al., 2014)). (c) Simultaneous scalp and intracranial subdural HFOs (adapted from (Zelmann et al., 2014)).

Pathological brain activity in patients with epilepsy is often detected in/on the cortex with intracranial depth or subdural electrodes, with no visible simultaneous abnormalities on scalp EEG. Some patients can have pharmaco-resistant epilepsy with only few or even no scalp-visible abnormalities between seizures. This can be due to geometrical characteristics of the cortical region of the irritative zone (the region generating interictal discharges), such as small extent, cortical folding with electric field cancellation, medial or basal cortex with insufficient scalp sampling to observe a characteristic voltage map. The extent to which scalp EEG can capture activity in these regions has long been the subject of controversy. A minimum cortical extent of 6 cm^2^ was originally proposed more than 50 years ago (Cooper et al., 1965) and has been the rule of thumb ever since. In line with this, initial simultaneous studies with scalp and subdural temporal electrodes showed that 90% of interictal epileptiform discharges (IEDs) involving cortical surfaces >10 cm^2^ were detected, compared to only 10% if <10 cm^2^ and none if <6 cm^2^ (Tao et al., 2005). In line with this, simulation work showed that the presence of subdural grids may attenuate the transmission of the underlying cortical activity and enhance transmission at its borders so that these surface measures were overestimated and a minimum of 4–8 cm^2^ of involved cortex could correspond to scalp detection thresholds (von Ellenrieder et al., 2014). Importantly, even when IEDs following the standard definition are not “detected” with standard 10–20 montage, in many cases pathological looking EEG patterns can be observed (Tao et al., 2005). High density scalp electrode (hd-EEG) with simultaneous iEEG sampling has only been reported in a small number of studies, showing correct lobular localization (Yamazaki et al., 2012, 2013)(Fig. 2.A).These studies have determined that purely temporal medial IED can be detected with a rate of 45% (Yamazaki et al., 2012). Involvement of basal temporal areas may be related to the high conductivity of the electrical field by skull base foramina allowing their maximal detection in anterior basal electrodes (Nayak et al., 2004). In addition, ideal and basal frontal regions, 4/29 frontal lobe intracranial IED types were detected on the scalp (Ramantani et al., 2014). Even insular IEDs, typically difficult to visualize on scalp EEG, could be detected automatically with a sensitivity of 52%, more so when the IED rate was higher (Iachim et al., 2021). Other studies have used validation with foramen ovale electrodes that can only lateralize IEDs and seizure occurrence in the medial/basal temporal lobe (Zumsteg et al., 2005). Moreover, even when visually undetected at individual level, IEDs have a scalp EEG fingerprint with small signal-to-noise ratio, that becomes apparent when intracranial IEDs are averaged not only for neocortical IEDs (Ramantani et al., 2014), but also for mesio-temporal ones (Koessler et al., 2015)(Fig. 2.B). In the latter study, there was a measurable scalp EEG signal for the hippocampal IEDs cluster (with a small signal in the mesial contact of the basal temporal electrodes, F9, P9, with amplitude 5–10 lower than for IEDs involving the lateral temporal cortex). Simultaneous recordings also reported an added value of nasopharyngeal electrodes (+25%) over cheek electrodes or anterior temporal electrodes for detecting medio-basal temporal IEDs (Zijlmans et al., 2008).

High Frequency Oscillations (HFOs; 80–500 Hz) have emerged as another interictal signature of epilepsy (Frauscher et al., 2017; Jacobs et al., 2012; Zijlmans et al., 2012). Given their small cortical extent of the order of millimeter and given the assumptions listed above, it was initially counterintuitive that HFOs could be recorded with scalp EEG. Simultaneous recordings with standard clinical montage (low-density) demonstrated the cortical origin of scalp HFOs (Zelmann et al., 2014) (Fig. 2.C). A follow up study investigated the prognostic value of simultaneous recordings, showing that widespread scalp HFOs was associated with poor post-operative outcome (Kuhnke et al., 2019). Conducting simultaneous recordings presents a valuable approach for elucidating the relationship between scalp HFOs and iEEG HFOs, particularly considering the scarcity of HFOs detected on EEG/MEG (van Klink et al., 2016; Zelmann et al., 2014). Analyzing HFOs using simultaneous recordings could aid in determining the optimal parameters in terms of spatial sampling, electrode montage (e.g., referential, bipolar, Laplacian), and preprocessing techniques (e.g., ICA strategies (Coelli et al., 2023), virtual sensors (van Klink et al., 2016)), to enhance their detectability non-invasively.

### 3.2. Detection, lateralization, and localization of seizures

Beyond detection and localization of interictal (in between seizures) activity, ongoing simultaneous scalp and intracranial EEG could add crucial insight about the dynamics of early ictal changes, ictal pattern that remain very focal, seizure propagation, and termination. Seizure onsets can sometimes be recorded intracranially several seconds before the occurrence of scalp ictal changes or clinical manifestations. Only a third of subclinical or focal aware seizures were detected with scalp EEG in a sample of 172 seizures recorded with simultaneous recordings (Casale et al., 2022). Some of the non-detected seizures could be mistaken for interictal activity. Scalp seizure detection was higher for non-lesional MRI despite shorter duration, highlighting the clinical relevance of EEG in non-lesional cases. This finding also suggests a wider network involved in epileptic activity in non-lesional cases and a more confined activity in lesional cases, potentially also involving more cancellation of electrical fields in complex lesion geometry (Casale et al., 2022). Furthermore, agreement in latency, lateralization, and ictal EEG patterns was associated with good post-operative outcome (Abramovici et al., 2018). Simultaneous scalp and foramen ovale electrodes also showed that unilateral medial seizure onset could manifest as bilateral scalp onset and scalp onset occurring after the first clinical manifestation still had good lateralization and localization (Alarcón et al., 2001). In studies using depth electrodes, subdural strips, and temporal scalp electrodes, seizures were only detected on the scalp EEG, after propagation from the hippocampal onset to parahippocampus and lateral neocortical structures (Pacia and Ebersole, 1997; Vossler et al., 2017). These as well as the needed discharge synchrony (Tao et al., 2007) present an apparent discrepancy with the possibility to detect purely medial IEDs with EEG/MEG (see Section 3.1). This paradox may be resolved when considering the lower signal-to-noise of individual ictal onsets compared to IEDs coupled with the analyses of individual seizures separately vs. averaged IEDs (individual purely medial temporal IEDs are also very difficult to detect). In addition, the absence of a full head electrode coverage of mid-to-high spatial density may also have contributed to this delayed detection. Indeed, an amplitude reduction of 1:100 was reported in one case of high-amplitude (1500–2000 μV) and highly synchronized purely hippocampal seizure detected on scalp EEG (Mukae et al., 2023).

Signal processing approaches could help identify subtle early ictal changes on scalp recordings. For example, decomposition of scalp EEG into independent spatio-temporal components (such as ICA) have shown that some components and their estimated cortical sources could represent a good signature of poorly visible early ictal alterations on scalp EEG (Barborica et al., 2021). This work was building up on a previous application to interictal simultaneous MEG-iEEG that some MEG components could represent temporal medial activity (Pizzo et al., 2019) that is otherwise poorly detected by MEG due to the rapid magnetic signal decay. Of note, such poor detection was also shown by simultaneous subdural iEEG and 37 MEG sensors applied locally over the intracranial recording (Shigeto et al., 2002).

In a subset of mesial TLE (from the dataset in Casale et al., 2022), deep learning allowed reliable classification of 1-second scalp EEG segments into interictal, pre-ictal, and ictal scalp EEG, even for visually undetected temporal seizures, with high accuracy (Li et al., 2021). Machine learning approaches were even able to identify scalp EEG segments with underlying hippocampal IEDs, although involvement of other brain structures could not be assessed with foramen ovale electrodes, with increased sensitivity compared to expert readers (Abou Jaoude et al., 2022). These approaches could be crucial in the identification of subtle epileptic manifestations potentially associated with worse cognitive evolution in patients with epilepsy and the identification of new biomarkers as described next.

### 3.3. Determining the pathological nature of other scalp EEG transients

The epileptic or physiological relevance of some transient patterns recorded with scalp EEG is ambiguous in some circumstances. In the following, we present five examples of how simultaneous iEEG helped disentangle whether scalp patterns were pathological or physiological.

#### Intermittent Rhythmic Delta Activity (IRDA).

**Fig. 3.**
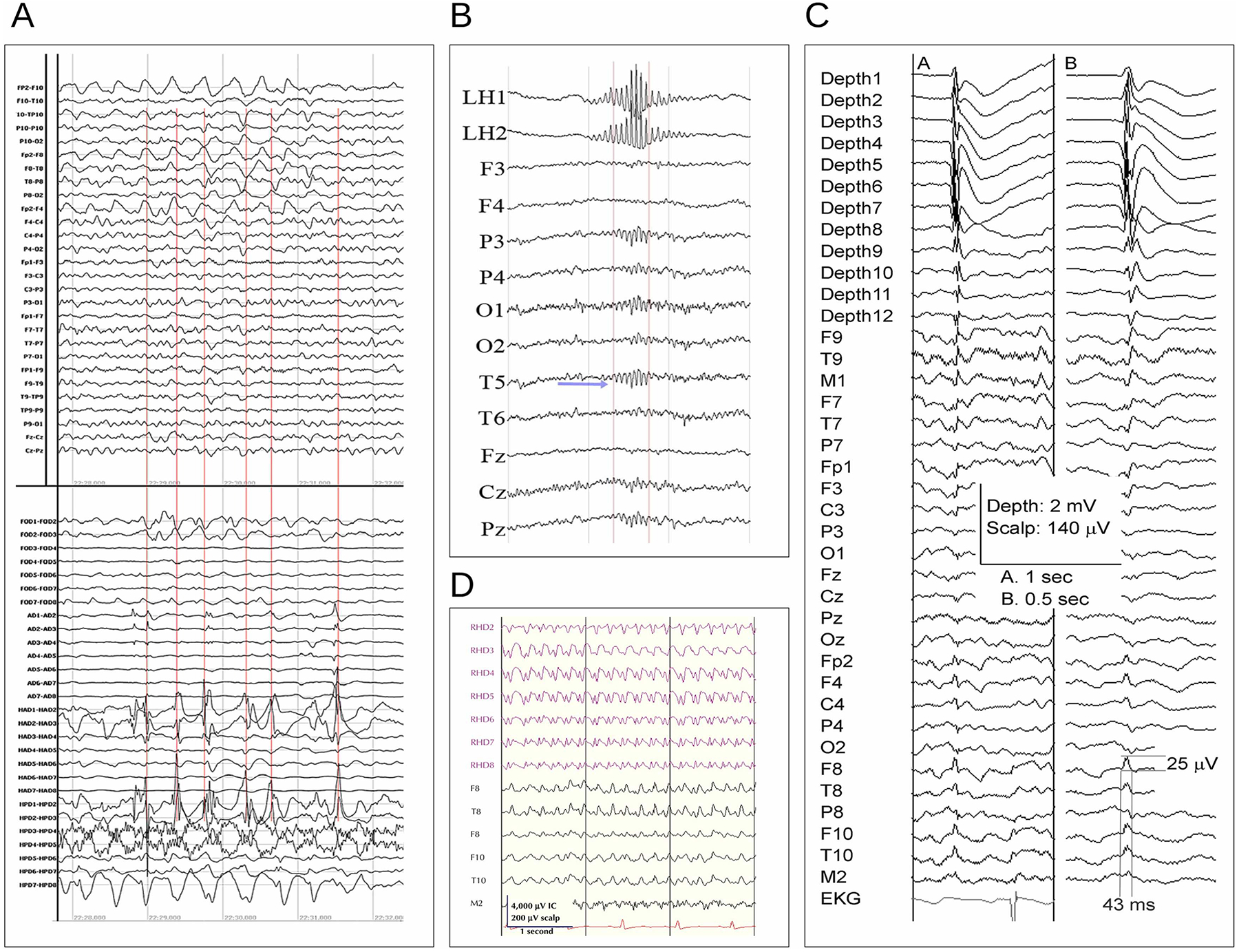
(a) Scalp EEG (bipolar montage) with 2.5 Hz LRDA in the right frontotemporal region and iEEG (bipolar montage) showing epileptiform discharges in the right hippocampus (HAD1–3 and HPD 1–3; adapted from (De Stefano et al., 2020)). (b) Manifestation of the 14&6/sec positive IEDs variant and its intracranial hippocampal correlate (adapted from (Kokkinos et al., 2019)). (c) BETS on scalp EEG co-occurring with intracranial hippocampal IEDs at two different time scales (adapted from (Issa et al., 2018)). (d) Right temporal scalp rhythmic temporal theta bursts of drowsiness (RTTBD) and right hippocampal ictal discharges (adapted from (Sun et al., 2020)).

Patients with temporal lateralized IRDA (TIRDA or LRDA) are more likely to suffer from epilepsy or acute asymptomatic seizures when critically ill (Gaspard et al., 2013), but what these patterns represent is an ongoing debate. Simultaneous recordings have shown, that IRDA observed on the scalp EEG, co-occurred with rhythmic cortical spike-waves recorded invasively (De Stefano et al., 2020) (Fig. 3.A) and that scalp EEG delta activity >1.4 Hz correlates with underlying intracranial interictal IEDs, in line with the 1.5 Hz cut-off corresponding to high vs. low association with seizures (De Stefano et al., 2022). These studies suggest the possible “epileptiform” significance of such transients devoid of scalp visible IEDs.

#### Non-pathological 14&6 spikes.

14&6/sec spikes on the contrary are an example of scalp pattern that might not be epileptic. In this case, simultaneous studies showed that scalp 14&6/sec spikes corresponded to “barques”, bursts of non-epileptic hippocampal activity, likely related to physiological sleep activity (Kokkinos et al., 2024, 2019)(Fig. 3.B).

#### Benign Epileptiform Transients of Sleep (BETS).

Conversely, simultaneous recordings offer the opportunity to explore the largely unexplained underlying mechanisms of activities with either normal EEG variants or of unknown significance. BETS (formerly called Small Sharp Spikes - SSS) patterns were traditionally considered benign when recorded on the scalp (White et al., 1977), but their cortical origin is not well understood. For instance, MEG/EEG source localization suggested that BETS have complex spatio-temporal dynamics in the hippocampus, potentially related to sleep processes (Wennberg et al., 2020). However, using simultaneous scalp and intracranial hippocampal recordings, two independent groups found them predominantly in the seizure onset zone, which suggests that they may be more pathological than often considered (Bruzzone et al., 2022; Epitashvili et al., 2021; Issa et al., 2018) (Fig. 3.C).

*Lateralized Periodic Discharges (LPD), and Rhythmic Temporal Theta Bursts of Drowsiness (RMTD)* are additional examples of brain activity of unknown significance, suggested to be related to intracranial pathological activity by recent simultaneous recordings. A simultaneous study found RMTD related to subclinical, sometimes even clinical seizures but their frequent occurrence suggests that they only partially overlap with the classical pattern considered to be a non-pathological variant and only rarely observed even in long-term EEG (Sun et al., 2020)(Fig. 3.D). Scalp LPD have likewise been associated with trains of IEDs in the medial temporal lobe or prolonged seizures (Sakata et al., 2022). This supports their increasing consideration as highly epileptiform patterns in the ictal-interictal continuum. In these cases, however, the periodic activity had a frequency lower than the 1 Hz and no coexistence of typical fast or rhythmic activity that would formally classify them in the interictal-ictal continuum in the critical care EEG classification (Hirsch et al., 2021).

These examples illustrate how simultaneous recordings could help in the classification of noninvasively recorded patterns as physiological or pathological, helping define electrographic biomarkers and understand their cortical correlates.

### 3.4. Combining iEEG and hd-EEG/MEG to improve clinical conclusions

Simultaneous scalp EEG/MEG iEEG mutually enhance each other. Not only can simultaneous recordings validate scalp observations using iEEG as the gold standard, as described above, but importantly scalp EEG/MEG can completed the inherent limited spatial sampling of iEEG.

For example, discrepancies between scalp EEG and iEEG, such as variations in seizure latency, morphological patterns, or topographic onset locations, were associated with a lower chance of detecting the seizure onset zone (Abramovici et al., 2018). These could indicate that the epileptogenic zone has been insufficiently sampled by intracranial electrodes. In line with this, in a simulation study of unilateral iEEG implantation and standard scalp EEG, simultaneous recordings allowed correct lateralization of seizures in 92% of cases compared to 33% with unilateral iEEG alone (Antony et al., 2019). These illustrates the powerful synergy of the two modalities, as the with scalp EEG offering an extended “whole brain” low-resolution picture complementary to the iEEG.

### 3.5. Source localization with simultaneous recordings could better delineate epileptic networks

Simultaneous recordings not only provide the most stringent form of validation for source imaging (Yamazaki et al., 2012), allowing to test the effect of spatial sampling, head models, inverse solutions on a specific set of IEDs, or even individual IED, but allows for a more powerful synergic approach to enhance source imaging methods. The larger spatial sampling offered by hd-EEG/MEG, augments the spatial coverage of the iEEG and could help improve source imaging methods, by weighting the inverse solution with iEEG priors or by integrating both modalities. As an example of iEEG priors iEEG-informed minimum-norm estimates method was proposed (Shu et al., 2022) and consisted in guiding the source reconstruction using IED locations observed on iEEG. This is achieved by modifying the source covariance matrix based on the proximity of the sources to the iEEG IED location and the estimated source power in the vicinity of the SEEG contacts. To account for unsampled regions, the source covariance matrix is further modified to enhance sources remote from iEEG electrodes strongly activated during the IED, as estimated by classical minimum norm estimate. Using both simulation and real data, this method was shown to generate more accurate source estimation than the traditional minimum-norm estimates method. Furthermore, it successfully unveiled IED locations that were overlooked by iEEG (Shu et al., 2022). An alternative is combining EEG and iEEG modalities during source imaging (i.e., by estimating the inverse solution of both modalities). A simulation study showed that source imaging using only iEEG data had limited efficacy in accurately localizing sources located more than 15 mm away from the depth electrodes and that accuracy could significantly improve with simultaneous scalp EEG and iEEG (Hosseini et al., 2018). Simultaneous MEG-EEG-iEEG could potentially further provide clinical information (Gavaret et al., 2016). In one patient, focal intracranial IED not visible on the surface were marked and source localization performed on a MEG average triggered on the iEEG markers. A dipole source was found in the primary visual cortex consistent with the seizure semiology. This region was posterior to the most posteriorly placed depth electrode that cannot be easily sampled invasively (Gavaret et al., 2016).

In summary, simultaneous iEEG and EEG/MEG recordings could be more than a pure research tool and provide clinicians with crucial complementary information, providing both specific cortical and subcortical localization while mitigating the limited spatial sampling of iEEG.

## 4. System neuroscience: complementing different views of the human brain by linking scales

### Scalp EEG and MEG are the standard method for millisecond resolution studies in system neuroscience.

Intracranial recordings in humans have been increasingly used as a window into the human brain, but as discussed above, its view is limited to where electrodes are implanted. To complement iEEG recordings and to generally advance our knowledge of brain function, researchers have begun to measure and analyze simultaneous recordings from intracranial and noninvasive modalities from the perspective of system neuroscience and general electrophysiology. This approach holds significant promise in providing a comprehensive understanding of local and distributed neural networks, the underlying mechanisms of brain function and dysfunction, and the nature of how brain information changes at different spatial scales. In this section we present some examples of this promising field.

### 4.1. Simultaneous recordings of spontaneous activity

As outlined above, simultaneous scalp and iEEG are shedding light on how deep brain activity (e.g. occurring in mesial temporal lobe) manifests at the scalp. Extending similar analysis to non-pathologic areas and periods of brain activity could consequently provide the foundation for building a more generalizable model for neural source localization of noninvasive activity. These models could be applied to better understand neural underpinnings and develop brain-based biomarkers for other neurological and psychiatric disorders.

#### Resting state connectivity.

Both MEG and hd-EEG are capable of capturing resting state networks similar to those obtained with fMRI (Brookes et al., 2011; Coquelet et al., 2020). Resting state activity recorded with iEEG provides a unique opportunity to study interactions between cortical and subcortical structures. By combining high-resolution neural activity captured by the extensive deep electrode placement in patients with iEEG coupled with simultaneous whole brain scalp EEG recordings, researchers can examine the dynamics of distributed networks involving regions that are not easily accessible through scalp EEG alone. This approach holds immense potential in elucidating the complex interactions between different brain regions and their contribution to overall brain function. A first step to relate these signals would be to understand the relationships between contemporaneous segments of intracranial and scalp EEG time series. Each of these time series have well-studied features that can be extracted, such as measurements of periodic or rhythmic activity (Buzsáki and Draguhn, 2004; Engel et al., 2001) and of phase and amplitude of aperiodic activity (Donoghue et al., 2020). These features can be extracted from both scalp and intracranial features and the relationship between each can be elucidated via regression analysis. Examples of these type of analysis have been studied during sleep and to a lesser extent during memory tasks (Section 4.3).

#### Understanding sleep physiology.

Scalp EEG during the intracranial investigation is clinically used to assess sleep stages, permitting also studying the physiological mechanisms of sleep signatures. Indeed, even though intracranially K-complexes are maximal in medial and lateral frontal lobe cortices, consistent with frontal midline scalp distribution (Wennberg, 2010), scalp K-complexes were shown to have widespread cortical distribution (Cash et al., 2009; Latreille et al., 2020). Scalp recorded slow waves could originate anywhere in the neocortex, with preference for frontal to posterior/temporal propagation (Botella-Soler et al., 2012). Scalp spindles also showed widespread cortical activation with low synchrony across the brain (Frauscher et al., 2015). Not only the neocortex, but also the role of deep structures, such as hippocampus (Frauscher et al., 2015) and amygdala (Muñoz-Torres et al., 2018), and their synchronization with the neocortex (Muñoz-Torres et al., 2023) could be unfolded. Moreover, the interplay between spindles and hippocampal sharp wave ripples (Buzsáki, 2015) is fundamental for memory consolidation (Girardeau and Lopes-Dos-Santos, 2021). The co-occurrence of hippocampal ripples with scalp spindles might help separate between physiological from pathological ripples (Bruder et al., 2021). Non-oscillatory mechanisms could also contribute to enhance long-term memory (Lendner et al., 2023). Thalamocortical oscillations mediated synchronization is fundamental for sleep homeostasis (Steriade and Timofeev, 2003). Recently, thalamic activity corresponding to sleep signatures recorded on the scalp EEG has been studied (Peter-Derex et al., 2023).

#### Source localization to relate non-invasive and invasive neuroscience studies.

One of the most challenging issues when using scalp EEG, both in neuroscience and in clinical practice, is to determine precisely where in the brain is generated the neural activity recorded from the scalp. To face this issue, in the last decades major algorithmic advancements like blind source separation methods as well as source reconstruction gave rise to the so-called “electrical neuroimaging”. However, the EEG inverse problem is ill-posed, i.e., its solution is non-unique and sensitive to noise and modeling errors (Miinalainen et al., 2019), and this is why the reconstruction process needs additional apriori constraints to make the model converge towards a solution (Brette and Destexhe, 2012). Moreover, it strongly relies on the accuracy of the EEG forward problem, i.e. the knowledge of the electric field generated at the scalp by any primary current source localized in the brain. In essence, this field suffers from the lack of a ground truth, i.e. a real solution to which any method should point to be verified.

When studying brain connectivity there are additional methodological concerns with the current inverse solutions as the majority of available inverse solutions have been optimized and validated to localize individual sources (Grova et al., 2006; Mikulan et al., 2020; Pascual-Marqui, 2002). Connectivity analysis requires not only that the sources are well localized but also that their time courses are correctly reconstructed. However, MEG and scalp EEG record a mix of signals that even after eliminating zero-lag correlations, are still influenced by interactions (Palva et al., 2018). Controlling how the signal of one source is affected by the signal of others has been shown to be the most crucial point for connectivity analysis (Colclough et al., 2015). Simultaneous recordings could permit, by providing a gold standard brain network (albeit limited to the sampled areas), to quantify and optimize inverse solutions in terms of accuracy of the time course reconstruction. Initial attempts corelated the MEG source imaging results to a forward model from iEEG of a later depth electrode implantation in the same patients, thus validating the origin of MEG source localization (Grova et al., 2016). An even better approach would be to use simultaneous MEG/scalp EEG and iEEG (López-Madrona et al., 2022).

Thus, one promise of generating large datasets of simultaneous intracranial and scalp EEG recordings is the ability to build generalizable models of cortical-subcortical connectivity and their dynamic interaction. Models of this variety would take noninvasive scalp recordings as input and infer the concurrent time series in a variety of deep brain regions, trained on the ‘ground truth’ information yielded from the iEEG. This approach is akin to solving the inverse problem in traditional EEG source localization (de Munck et al., 1988; Pascual-Marqui, 1999). Thus, the development of such models would aid in our understanding of how subcortical sources manifest on the scalp, which can then be applied to healthy participants or patient populations that do not undergo surgery for electrode implantation. These predicted intracranial brain states could then offer richer information on their relationship to a patient’s disease pathology or symptom trajectory and could help translate the vast literature on noninvasive resting state and sleep to their intracranial correlates.

### 4.2. Simultaneous recordings during intracerebral electrical stimulation

Since Penfield’s seminal mapping of the homunculus (Penfield and Boldrey, 1937), stimulation with simultaneous intracranial EEG recordings has become invaluable as a research and clinical tool. SPES combined with invasive iEEG recordings is a powerful clinical tool for mapping pathological activity during presurgical evaluation of epileptic patients (Matsumoto et al., 2017; Valentín et al., 2005). It is also considered the gold standard for studying the effective connectivity of the human brain (Trebaul et al., 2018). However, intracranial recordings are spatially sparse as their topology are exclusively driven by clinical needs (Gavaret et al., 2016). This sparsity hampers systematic comparisons across-participants, the detection of the whole-brain spatiotemporal properties of the Cortico Cortical Potentials (CCEP) evoked by Single Pulse Electrical Stimulation (SPES), as well as their comparison with typical sensory evoked potentials.

**Fig. 4.**
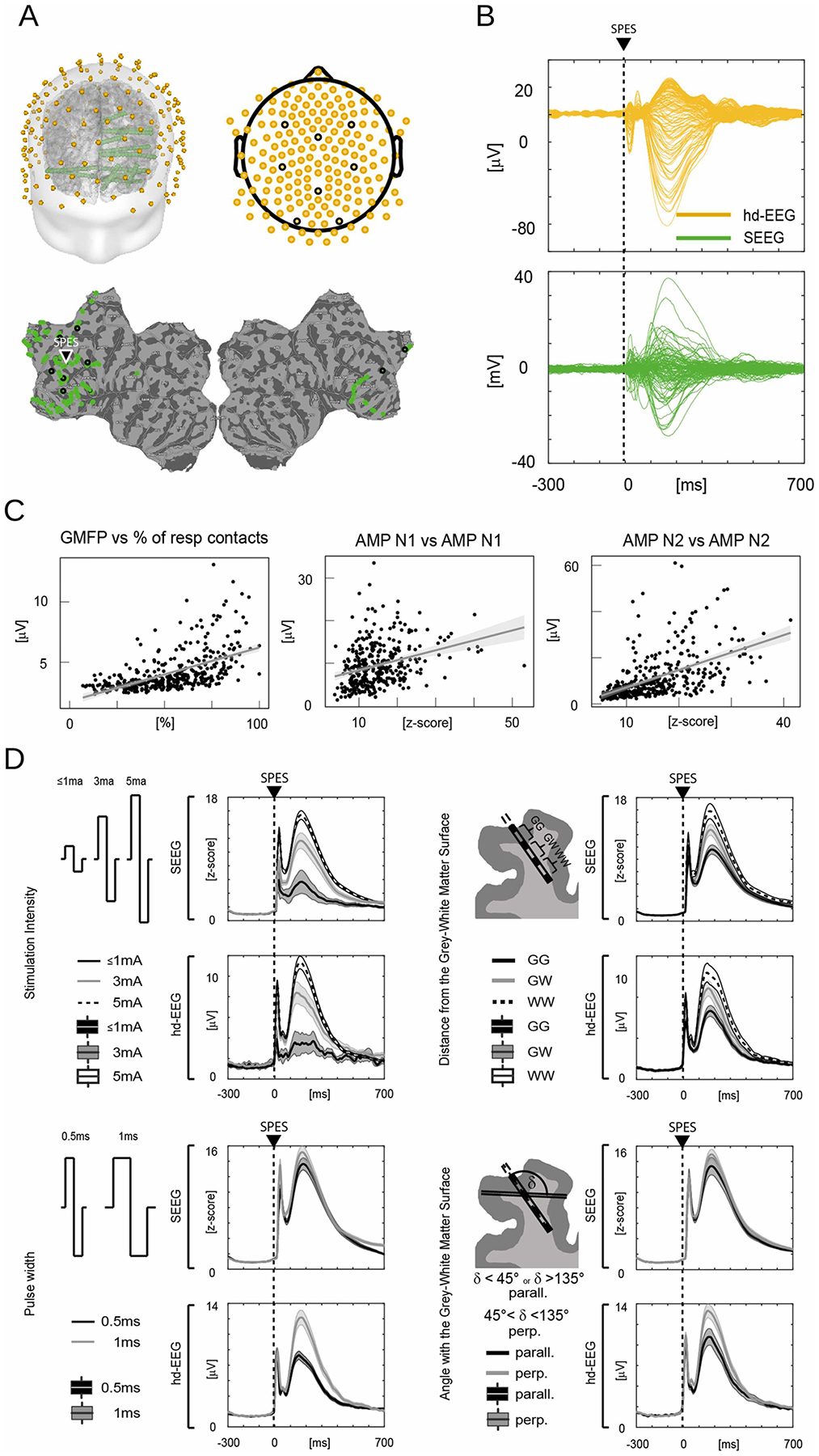
Simultaneous stereo-EEG and high-density scalp EEG recordings to study the effects of intracerebral stimulation parameters. A. 3D reconstruction of the position of intracerebral (green) and hd-EEG contacts (yellow). B. Butterfly plot of one representative stimulation session, recorded from the scalp (yellow) and with intracerebral electrodes (green). C. From left to right, linear regression between GMFP calculated at the hd-EEG level and the number of SEEG contacts responding to SPES with a significant CCEP (r = 0.592, p < 0.001); linear regression between the amplitude of N1 component calculated for both hd-EEG and iEEG (r = 0.313, p < 0.001); linear regression between the amplitude of N2 component calculated for both hd-EEG and iEEG (r = 0.553, p < 0.001). D. Global Mean Field Power (GMFP) of the hd-EEG and iEEG responses to SPES delivered at different physical and geometrical stimulation parameters (adapted from (Parmigiani et al., 2022)).

To overcome this limitation, a recent study recorded simultaneously hd-EEG (256 channels) and iEEG during SPES (Fig. 4; (Parmigiani et al., 2022). The dataset (available online at: https://doi.org/10.17605/OSF.IO/WSGZ) includes CCEPs collected from 36 epileptic patients encompassing different stimulation parameters which were lumped in three categories: physical (stimulation intensity and pulse width), geometrical (position of the bipolar contact with respect to grey/white matter and the angle of the electrode with respect to the cortical surface), and topological (stimulated cortical area). A combined analysis of intracerebral and scalp CCEP showed that: 1) overall invasive and non-invasive CCEPs are generally correlated (Fig. 4C); 2) differences in pulse width, angle and stimulated cortical area are on average better captured by hd-EEG (Fig. 4D); 3) hd-EEG responses to SPES reproduce basic features of non-invasive stimulation and recording assessments (e.g., transcranial magnetic stimulation - TMS - and EEG); 4) CCEPs, although they are not consciously perceived, show a much larger amplitude as compared to typical sensory evoked potentials and with those evoked by non-invasive stimulation (Parmigiani et al., 2022).

These results are important because they demonstrated the complementarity of invasive and non-invasive recordings in capturing the brain response to external perturbations. Moreover, they represent the first step toward filling the gap between invasive and non-invasive stimulation techniques. Along the same line, this gap has been recently further shortened by Wang et al. that recorded the intracranial response to TMS, for the first time in humans (Wang et al., 2024).

Another unique advantage given by the simultaneous recording of invasive and non-invasive EEG during intracerebral electrical stimulation consists in providing a ground-truth for source localization methods (Mikulan et al., 2020; Pascarella et al., 2023; Sun et al., 2023). The simultaneous recordings of scalp-EEG and iEEG during intracerebral electrical stimulation represent a unique opportunity to try to solve some of the methodological issues of source imaging. In other words, when we inject current (via SPES) through specific intracerebral contacts and we record from the scalp the EEG activity we provide the “solution” of the “inverse- problem”- i.e. a ground-truth for testing methods for source and forward modeling. As an example of the importance of sharing this unique data, the recent datasets from Mikulan and colleagues (Mikulan et al., 2020) has already been used to test and compare different source modeling methods (Pascarella et al., 2023; Sun et al., 2023).

### 4.3. Cognitive processes captured by recordings at different scales

By contrast with the large responses evoked by direct electrical stimulation such as SPES and with spontaneous epileptiform activity often involving large cortical patches exhibiting hyper-synchronized activity, both of which have good detectability on the scalp or using MEG, cognitive processes evoke more subtle activations, involving deep brain structures, and high-frequency activity. Thus, localizing the brain regions involved in cognition using scalp EEG or MEG is challenging. Simultaneous recordings may bring essential insights to bridge the gap between non-invasive large population cognitive studies and small population iEEG ones. A prime example of the advantage of using simultaneous recordings is the understanding of memory processes, as the hippocampus is known to be the key player in animal and invasive studies, but its signals cannot be easily recorded with non-invasive means. For example, three recent studies have analyzed the visibility of memory-related activity using simultaneous MEG-iEEG (López-Madrona et al., 2022) and scalp EEG-iEEG (Barborica et al., 2023; Ye et al., 2022).

To identify and separate the different brain sources that contribute to the MEG recordings, second-order blind identification (SOBI) (Belouchrani et al., 1997, 1993; Tang et al., 2005), that takes advantage of the temporal correlation within sources, has been used by López-Madrona et al., (2022). The topography of the independent component identified by SOBI that is common to all participants, putatively associated with memory processes based on the timing and significant differences between novel and familiar stimuli, is dipolar and the location of the associated bilateral dipoles points to a mesial activation. The validation of this finding, derived from non-invasive MEG recordings, has been performed by calculating partial correlation with simultaneous iEEG. The contacts located in rhinal cortex, hippocampus and to a lesser extent the middle temporal gyrus, exhibited the highest correlation between SOBI-MEG and iEEG. These findings demonstrate that blind-source separation methods applied to MEG surface recordings are capable of revealing deep mesial memory-related activations (López-Madrona et al., 2022).

**Fig. 5.**
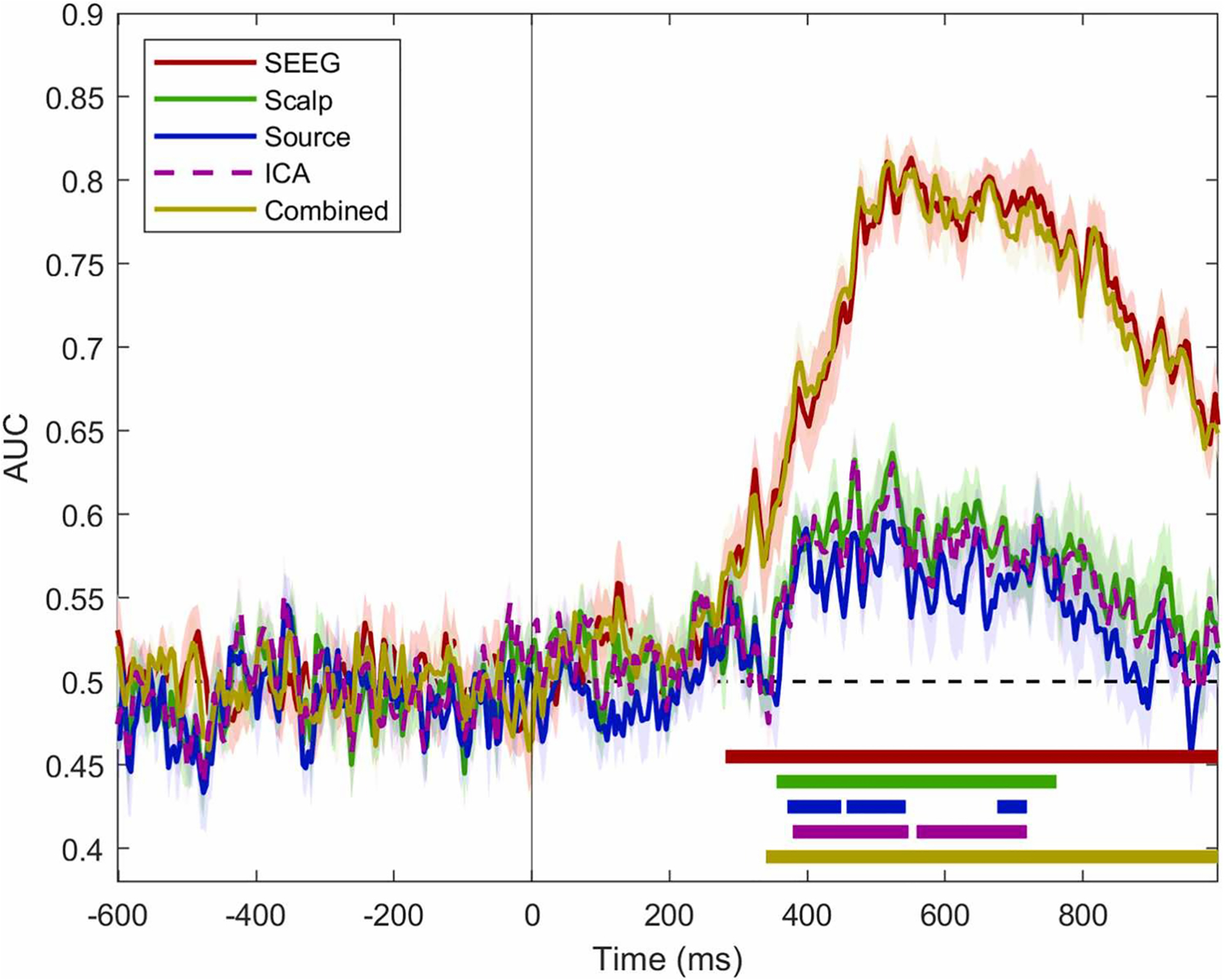
Multivariate pattern analysis (MVPA) classifier’s performance scored using receiver operator characteristic (ROC) area under curve (AUC), for iEEG, scalp EEG, source, ICA, and combined scalp-iEEG signals (n = 12 participants) performing a recognition memory task requiring distinguishing between novel and familiar visual stimuli. The dashed areas show standard error intervals. The horizontal bars indicate the intervals where the scores obtained following a 20-fold cross-validation are statistically different from chance (one-sample permutation cluster test, p < 0.05) (reproduced from (Barborica et al#, 2023)).

Alternatively, given the low amplitude of the evoked scalp responses during cognitive tasks, high-sensitivity methods like MVPA (Grootswagers et al., 2017; Haxby et al., 2001) allowed comparison of the performance of a linear classifier in decoding task conditions over the course of a memory recognition task (Besson et al., 2012; Despouy et al., 2020; López-Madrona et al., 2022) for scalp EEG, iEEG, as well as information derived from scalp recordings following ICA and beamformer source reconstruction (Barborica et al., 2023)(Fig. 5).

When comparing individual recording techniques, iEEG outperformed scalp EEG, even after ICA or beamformer, in decoding task conditions, despite the limited spatial sampling of the brain with depth electrodes. This may be related to the limited scalp visibility of the activity related to memory processes taking place in deep brain structures and to the different signal-to-noise ratios of the two recording modalities. By pooling together the scalp and intracranial EEG recordings, the decoding performance of a classifier using this superset was not higher than the modality exhibiting highest scores, iEEG, indicating that in this case there was little synergy between the two modalities (Barborica et al., 2023).

To further understand the relation between subcortical regions and scalp recordings, phase amplitude coupling (PAC) could also provide interesting insight into different frequency band contributions. For example, during a verbal memory task, the amplitude of iEEG high-gamma components was associated with the phase of low-frequency components in scalp EEG (Ye et al., 2022).

These examples exemplify how simultaneous scalp EEG/MEG and iEEG recordings advance our understanding of the similarities and differences measured by individual approaches and how combining them could advance our understanding of human neuroscience. We anticipate that the number of cognitive studies utilizing simultaneous recordings will continue to increase.

## 5. Challenges and future directions

The metaphor of the brain speaking in hieroglyphs and the need for a novel Rosetta Stone to translate it is particularly true in the case of the human brain, in which single neuron activity is recorded only exceptionally, and thus we can only “listen to its echo” from outside the skull with non-invasive methodologies. Given this constraint, one could argue that animal models would better serve the cognitive neuroscience scope, similarly to what happened for pharmacology or basic neurophysiology. This is particularly true for invasive recordings in primates which could offer a bridge between extensive single unit studies in animals and intracerebral explorations in humans. For example, (Jerbi et al., 2010) demonstrated the feasibility and utility of iEEG in elucidating cortical dynamics during cognitive tasks in macaque monkeys, providing a valuable avenue for comparative neurophysiological research; (Ray et al., 2008) investigated neural correlates of high-gamma oscillations (60–200 Hz) in macaque local field potentials, shedding light on the spatiotemporal dynamics of cortical activity that iEEG can capture; and (Bola et al., 2018) used iEEG in macaques (and humans) to study loss and recovery of consciousness in anesthesia.

Despite all this evidence, the human brain possesses almost exclusive abilities that prevent animal models from being a valid experimental platform, such as language (Gallardo et al., 2023), the capacity to instantiate, imitate, and encode complex manual abilities (Del Vecchio et al., 2020) including intransitive gestures (Subiaul, 2016), and the distinctive property of human self-awareness (Koch et al., 2016).

Given the premises above, we can derive that the human brain is the best model to study… the human brain, and iEEG offers an outstanding and privileged perspective. iEEG provides precise anatomical insights into how specific groups of neurons become active at a millimeter scale, while also revealing the temporal dynamics of their activation at a millisecond scale (Avanzini et al., 2016). When multiple nodes within a particular network are simultaneously monitored using implanted electrodes, iEEG signals can provide valuable information about functional interactions occurring within and between networks at various stages of neural processing. In this context, iEEG can help us advance our understanding of the human brain by providing novel insights not attainable through non-invasive human imaging or invasive recordings in nonhuman mammalian brains. Still, iEEG research has intrinsic limitations (Parvizi and Kastner, 2018), that could be complemented at least in part with simultaneous scalp EEG. For example:

1. *iEEG is recorded in specialized clinical settings*. iEEG is feasible only in clinical settings at selected hospitals, requiring specialized teams of clinicians and investigators. It is important to always remember that the priority is to obtain relevant clinical information and that research is secondary. Research participants in this setting have some level of pathology and the clinical environment constrains experiments, resulting in fewer trials and simplified designs. Moreover, electrode locations are clinically determined and cannot be altered post-implantation, in contrast to animal experiments. These restrictions severely limit accessibility, experimental design, and generalizability. The use of simultaneous scalp EEG/MEG could allow extrapolating results from intracranial investigation to non-invasive general population studies performed outside the clinical setting.
2. Sampling is sparse and heterogeneous, as we only see brain activity where electrodes are located. Electrodes are placed for clinical purposes and therefore certain brain areas, such as parietal, occipital, and inter-hemispheric regions, are less frequently implanted compared to frontal or temporal regions. Even in areas covered by electrodes, they are often positioned 5–10 mm apart, preventing more granular brain activity sampling. Moreover, subcortical regions including the basal ganglia, brainstem, and cerebellar areas are typically not monitored with iEEG due to a lack of clinical motivation, although recently thalamic implantation is becoming increasingly common (Gadot et al., 2022). Simultaneous scalp EEG/MEG could provide coverage of the whole brain and complement the specific location of depth electrodes and grids. The combination thus provides both full coverage and focal specificity.
3. *Neuronal Population Activity*. Due to SEEG and ECoG clinical macroelectrode size (surface of ~1–5 mm^2^), they average neuronal signals from a relatively large and diverse population of cells, more granular than scalp EEG/MEG (see for example (Miller et al., 2009)) but less granular than microelectrode recordings. In Section 5.3, we present some advances that could in the future allow truly multi-level spatial understanding, from scalp to macro to microelectrode recordings in the same participants. Most of the technical aspects presented in this review, such as synchronization and preprocessing, as well as neuroethical boundaries and data sharing apply to all these scales.

In this narrative review, we focused on how combining iEEG with non-invasive hd-EEG or MEG recordings could overcome some of these limitations offering a transfer function from inside to outside the brain. It is worth noting that these studies are only the beginning. Below, we discuss major future challenges that, when solved, will enhance the capabilities of simultaneous invasive and non-invasive recordings as a research and clinical tool.

### 5.1. Back and forth from population to individual level

As detailed throughout this review, iEEG provides an unparalleled view of brain activity dynamics. On one hand, iEEG has played a crucial role in treating drug-resistant focal epilepsies, especially when non-invasive recordings struggle to localize the affected areas (Cardinale et al., 2019; Isnard et al., 2018). In this framework, the approach resides at the individual level, as each patient is treated individually to identify the epileptogenic zone and the territories through which the ictal activity propagates during seizures. On the other hand, iEEG has emerged as the gold standard for studying brain functions due to its exceptional spatiotemporal resolution (Lachaux et al., 2012; Mercier et al., 2022), which is essential for modeling processes operating at subsecond scales. In this latter scenario, researchers often pool data from multiple patients to overcome individual sampling limitations (Avanzini et al., 2016; Kadipasaoglu et al., 2015), gaining comprehensive insights into brain dynamics and connectomics (Del Vecchio et al., 2021; Trebaul et al., 2018).

Despite their seemingly distinct domains—clinical utility versus neuroscientific insights—there is substantial value in the synergy between these approaches, which refines iEEG conclusions in clinical and cognitive/system neuroscience. Intracranial EEG primarily gathers data from patients with focal epilepsy, prompting the need to differentiate between healthy and pathological brain regions. Integrating clinical metadata is crucial for recognizing normal physiological information and understanding cognitive processes. Concurrently, treatment and resective surgical planning for epileptic patients demands a clear understanding of the physiological activity of the regions implicated in initiating ictal and interictal discharges. Only a population-level perspective can provide a sufficiently robust understanding, later tailored to individual patients for personalized insights. Ideally, centers performing intracranial recordings would benefit of the creation of tools integrating this information, possibly incorporating also of non-invasive simultaneous recordings.

Such a comprehensive approach opens to another fundamental aspect of neuroscience, i.e., the investigation of the variability of electrophysiological responses. Indeed, the low signal-to-noise ratio in most non-invasive recordings typically leads researchers to average signals across trials, thus treating the variability within- and between- patients as a variable of non-interest. The higher resolution of iEEG allows researchers to reverse this paradigm, investigating for instance the neuroanatomical, connectomics, or functional bases underlying activities deviating from population-based findings (Lachaux et al., 1999). The potential of this comprehensive (individual-to-population and back) approach is even more powerful if we include simultaneous iEEG with non-invasive recordings. Indeed, iEEG could help explain the variability in EEG, MEG (or fMRI, see below) responses and whether it depends on anatomical (e.g., the bias introduced across patients in the anatomical coregistration to the brain template), connectional (e.g., different connectivity with a given brain region), or functional features (e.g., scalp responses could reflect evoked or induced activity).

In light of these points, a challenge lies in crafting comprehensive tools that can seamlessly amalgamate all iEEG data, incorporating clinical insights, epilepsy biomarkers, and recordings during various states (resting, perceptual, and cognitive tasks), and pair with simultaneous non-invasive recordings. These integrated tools would form a versatile platform, enabling clinicians to analyze individual patient data within the broader context of other types of metadata. Simultaneously, they will empower researchers to authenticate and validate population-level findings at an individual patient level, fostering a more nuanced understanding of brain dynamics and personalized treatment approaches.

### 5.2. Widening the observation scale

As mentioned above, even though iEEG recordings are capturing signals directly from the brain, current clinical electrodes are registering activity from thousands if not hundreds of thousands of neurons. Since neurons and their synaptic interactions form the minimal unit for driving both pathological and normal brain function, iEEG and MEG/EEG can only offer the massive population-level view of neuronal activity. Work to capture single cell activity with the use of microelectrodes or fine microwires with simultaneous iEEG and low density EEG in the past two decades, though, has allowed us to span those spatial scales from a few hundred micrometers to centimeters of cortical recordings using iEEG (Cash and Hochberg, 2015; Chari et al., 2020; Paulk et al., 2024). These advances were made possible not only with the use of now FDA-approved neural recording devices such as the 100-channel microelectrode Utah array (Blackrock Microsystems), but also with the Behnke-Fried SEEG electrodes (AdTech, (Fried et al., 1999)) and the Dixi micro-macro hybrid electrodes (Dixi Medical) (Despouy et al., 2020, 2019), both of which have fine protruding microwires or tetrodes along with clinical macro-contacts for semi-chronic recordings during clinical monitoring for seizures (up to 29 days). These devices have been shown to be relatively safe for implantation (Carlson et al., 2018; Despouy et al., 2020), even, for the Utah array, for years (Rubin et al., 2023). Other devices such as laminar electrodes recording activity across the cortical layers in a focal area have also been used to detect and understand epileptiform activity along with cognitive processes (Fabo et al., 2023; Ulbert et al., 2001; Wang et al., 2005). Importantly, single cell recordings paired with the iEEG local field potential has offered crucial insight into the behavior of different neural subtypes (such as excitatory and inhibitory cells) during seizures and IEDs, revealing how seizures arise from underlying neural activity (Agopyan-Miu et al., 2023; Keller et al., 2010; Schevon et al., 2012; Truccolo et al., 2014, 2011). Indeed, multi-scale comparisons from the single cell to the ECoG or SEEG level are possible by pairing microelectrode and macroelectrode (clinical) iEEG recordings. However, aside from confirmation of seizure onsets using additional scalp leads by the clinical team, comparisons of simultaneous single cell recording activity with noninvasive signals are rare (Boran et al., 2019; Kubska and Kamiński, 2021; Topalovic et al., 2023), which is, unfortunately, a massively missed opportunity. Bridging the gap from single neural activity to recorded scalp dynamics may be made possible through the currently more common practice of sharing microelectrode data (Faraut et al., 2018), although simultaneous scalp data may also need to be made available. Alternatively, the data could be re-analyzed in groups who regularly perform these types of recordings where scalp signals are already recorded along with single cell activity.

On the other end of the spatial scale, there has been considerable work to bring together fMRI and iEEG/MEG recordings (Cunningham et al., 2012; Mukamel and Fried, 2011). Indeed, fMRI could provide the high spatial resolution and across-population level comparisons that are limited with individualized iEEG signals. This complementary information was highlighted in one simultaneous iEEG and fMRI study (Vulliemoz et al., 2011), where networks of BOLD changes correlated to intracranial epileptic spikes highlighted regions not sampled by the iEEG, results which have been reflected by further studies (Chaudhary et al., 2021; Sharma et al., 2019). Such datasets could also provide new insight regarding network connectivity, motor and cognitive tasks across recording modalities. Further, simultaneous fMRI and iEEG direct electrical stimulation is possible within safe limits (Fujita et al., 2022; Jones et al., 2014; Oya et al., 2017), with some data available for public use (Thompson et al., 2020).

An interesting challenge will be how to map the time scales from single cell activity to whole brain dynamics. With data collected across centers publicly shared it would be possible to explore the multi-scale comparisons from single cells to iEEG to EEG/MEG to fMRI in an exciting and rare opportunity that could provide fundamental mechanistic insight into human brain function and brain pathologies.

### 5.3. Neuroethical considerations

The combination of non-invasive and invasive brain recordings implies a few ethical considerations that are worth considering in order to maximize its potential clinical and scientific impacts. We here provide a summary of these considerations, while their analytical assessment is beyond the scope of this paper.

Invasive procedures in particular raise a number of ethical issues, as recently reviewed by (Feinsinger et al., 2022). They elaborate on two overriding ethical commitments to make invasive recordings ethically acceptable: maintaining the integrity of clinical care and ensuring the voluntariness of participation. The operationalization of these principles is not easy, because the actual clinical procedure is not consistent, particularly regarding recruiting and consent methods (Mergenthaler et al., 2021). While this lack of consensus may be justified in light of the innovative technology used, it may also be a stumbling block against its clinical exploitation. To advance towards an ethical standardization, it is crucial to first agree on the priorities to be assessed. A relevant proposal in this direction comes from (Chiong et al., 2018) who identify two dimensions of ethical scrutiny: invasiveness of methods used and modification of interventions for research aims. These two dimensions may work as heuristics for more fine-grained analysis.

In general, there are both potential ethical benefits and risks related to the combination of non-invasive and invasive brain recordings. Among the first better diagnostic and prognostic biomarkers as well as more precise therapeutic targets eventually leading to more effective clinical treatments. Therefore, since it provides more reliable information about the participant’s brain functions, there are ethical reasons for supporting the combination of non-invasive and invasive brain recordings. Also, this combination promises to make possible more targeted health treatments, in the direction of the personalization of medical interventions. Thus, it seems ethically justified to facilitate the clinical translation of research on invasive brain-recordings (i.e., to increase their use in clinical settings).

Among the risks to be considered are situations when the distinction between research and therapy is not easy to make (Chiong et al., 2018). Especially when the clinician is also the investigator, there is the risk of potential role conflict, more specifically of undue influence on the patient, whose consent is eventually not completely free (e.g., they might wrongly think that the provision of healthcare depends on their participation to research). Also, the risk arising from research or clinical use of invasive brain-recordings should be proportionate to the benefits for the participant involved. As the research participants are also patients, it is necessary to indicate in consent forms and protocols that there is no expected direct benefit deriving from their participation in research to prevent therapeutic misconception (Appelbaum et al., 1982).

Finally, there are some more procedural ethical issues that deserve specific attention, including the need for assuring a valid informed consent by patients (even in case of impaired cognitive capacity), the need for guaranteeing reliable data through adequate standardization, validation and anonymization procedures, the need for equal access to deriving clinical applications, and the need for open access data and research tools.

### 5.4. A call for standards, open data and software

iEEG data is inherently rare, and simultaneous recordings of iEEG with hd-EEG or MEG are even rarer. In the near future, it will be crucial to collect this data, structure it into datasets, curate the metadata, and openly share it, thus creating a valuable resource for method developers. To succeed in this challenging task, all research centers should collaborate to converge on standardized methods for organizing simultaneous data and finding common strategies to promote data sharing. For instance, the implantation of iEEG varies across centers, with some using cortical grid and strip electrodes, while others rely exclusively on stereotactic depth electrodes or a combination of both approaches. Achieving a comprehensive understanding of the entire brain and validating methods for each region necessitates close collaboration between centers, especially to gather sufficient data from rarely sampled regions, such as the occipital and parietal lobes. Fortunately, the Brain Imaging Data Structure (BIDS)(Gorgolewski et al., 2016), a community driven effort to standardize imaging data, already has specifications for iEEG (Holdgraf et al., 2019), EEG (Pernet et al., 2019) and MEG (Niso et al., 2018). Instead of requiring the creation of specific rules for simultaneous recordings, it would be advantageous to leverage the existing BIDS effort in organizing simultaneous data. Several tools exist to facilitate the organization of iEEG the data into BIDS (Appelhoff et al., 2019; Roehri et al., 2021; Zwiers et al., 2022); unfortunately, open software dedicated to packaging iEEG/hd-EEG/MEG databases into the BIDS format still do not exist. They would streamline the process of structuring iEEG/hd-EEG/MEG data, thus encouraging researchers to adopt standardized practices for data organization and sharing. Moreover, by providing a comprehensive solution for database packaging, researchers can focus more on data analysis and interpretation, ultimately accelerating scientific discoveries in iEEG/hd-EEG/MEG research.

Both data and software can be shared on dedicated platforms (e.g., OpenNeuro, Zenodo, FigShare, DABI) and researchers can gain recognition by describing their dataset in specialized journals (e.g., Scientific Data, Data in Brief). An important aspect that should always be considered when sharing human data is the protection of private information. Using strict de-identification and standardization (Mikulan et al., 2021) is crucial to remain within neuroethical bounds. Within these neuroethical boundaries, large and open shared datasets could help demonstrate the clinical utility of simultaneous recordings which in turn will allow expanding the adoption of simultaneous recordings in more centers, rather than perceiving them solely as research tools. This shift in perspective can contribute to an increased volume of simultaneous recordings being performed, simultaneously improving clinical care and neuroscience research.

## 6. Conclusions

Here, we reviewed the converging paths that led us to integrate intracranial and non-invasive human brain recordings. We have also delineated future work to help us develop new research and clinical tools grounded in simultaneous invasive and non-invasive recordings. As a community of neuroscientists and clinical researchers, each with a distinct focus yet united by a shared reliance of iEEG, we firmly believe that the integration of invasive and non-invasive recordings, coupled with collaborative initiatives across research centers will be key to deliver a novel Rosetta Stone to understand the language of the human brain.

## Data Availability

No data was used for the research described in the article.
